# Inter-Tributary Movements by Resident Salmonids across a Boreal Riverscape

**DOI:** 10.1371/journal.pone.0136985

**Published:** 2015-09-17

**Authors:** Kale T. Bentley, Daniel E. Schindler, Jonathan B. Armstrong, Timothy J. Cline, Gabriel T. Brooks

**Affiliations:** 1 School of Aquatic and Fishery Sciences, Box 355020, University of Washington, Seattle, Washington, United States of America; 2 Wyoming Cooperative Fish and Wildlife Research Unit, Department of Zoology and Physiology, University of Wyoming, Laramie, Wyoming, United States of America; 3 Fish Ecology Division, Northwest Fisheries Science Center, National Marine Fisheries Service, National Oceanic and Atmospheric Administration, Seattle, Washington, United States of America; University of California, Berkeley, UNITED STATES

## Abstract

Stream-dwelling fishes inhabit river networks where resources are distributed heterogeneously across space and time. Current theory emphasizes that fishes often perform large-scale movements among habitat patches for reproduction and seeking refugia, but assumes that fish are relatively sedentary during growth phases of their life cycle. Using stationary passive integrated transponder (PIT)-tag antennas and snorkel surveys, we assessed the individual and population level movement patterns of two species of fish across a network of tributaries within the Wood River basin in southwestern Alaska where summer foraging opportunities vary substantially among streams, seasons, and years. Across two years, Arctic grayling (*Thymallus arcticus*) and rainbow trout (*Oncorhynchus mykiss*) exhibited kilometer-scale movements among streams during the summer growing season. Although we monitored movements at a small fraction of all tributaries used by grayling and rainbow trout, approximately 50% of individuals moved among two or more streams separated by at least 7 km within a single summer. Movements were concentrated in June and July, and subsided by early August. The decline in movements coincided with spawning by anadromous sockeye salmon, which offer a high-quality resource pulse of food to resident species. Inter-stream movements may represent prospecting behavior as individuals seek out the most profitable foraging opportunities that are patchily distributed across space and time. Our results highlight that large-scale movements may not only be necessary for individuals to fulfill their life-cycle, but also to exploit heterogeneously spaced trophic resources. Therefore, habitat fragmentation and homogenization may have strong, but currently undescribed, ecological effects on the access to critical food resources in stream-dwelling fish populations.

## Introduction

River networks are hierarchically structured systems characterized by a continuum of downstream changes in biota and ecosystem processes, which generate coarse-scaled patterns of biotic and abiotic heterogeneity [1,2]. However, fluvial systems also exhibit high levels of finer-scale variation within these large-scale longitudinal patterns [3–5]. For example, habitat heterogeneity across an intact floodplain, or among tributaries, may be similar in magnitude to that across the entire river continuum [4,6] providing a patchily distributed mosaic of habitats for organisms whose resources vary in across space and time [7,8]. Ultimately, individuals must navigate and exploit the heterogeneity of their environment to successfully grow and reproduce. For aquatic ecosystems, our understanding of how mobile organisms exploit habitat networks remains distinctly incomplete.

Movement among habitats is one strategy animals have evolved to track spatially and temporally variable resources. For freshwater fishes, it has been widely acknowledged that relatively long-distance movements are necessary for the long-term persistence of populations [9]. However, the emphasis has mostly been on the need for large-scale, inter-seasonal movements to connect complementary habitats used for reproduction, refugia, and feeding [7,10,11]. Less attention has been paid to characterizing fish movements at the multi-kilometer scale within a season that may allow individuals to exploit the heterogeneity in feeding and growth opportunities that complex riverscapes [sensu 12] provide to them [e.g., 13,14]. In terrestrial ecosystems, trophic resources display substantial intra-seasonal variation across the landscape requiring animals to make large-scale movement to achieve positive energy balance [15–17]. We hypothesize that analogous to terrestrial studies, resident fishes may need to integrate across large-spatial scales when foraging opportunities are patchily distributed in space and time across river-networks. Here, we investigated the summer movement characteristics of stream-dwelling salmonids throughout a network of lake tributaries whose productivity in producing prey for fishes can vary substantially among streams and years.

Arctic grayling (*Thymallus arcticus*) and rainbow trout (*Oncorhynchus mykiss*) are two species of non-anadromous (resident) salmonids that co-occur in streams and rivers throughout western Alaska. Grayling and rainbow trout play a large ecological and economic role in the region, as they can often comprise the majority of resident stream biomass and are the mainstay of recreational fisheries [18]. The high productivity of these resident fish populations is supported by the annual return of spawning anadromous sockeye salmon (*O*. *nerka*). Specifically, the eggs of spawning salmon offer a high-quality resource pulse of food for resident fishes [19,20]. Prior to the arrival of salmon, the diet of resident fishes primarily consists of benthic and terrestrial invertebrates [19]. Once anadromous salmon begin spawning, grayling and rainbow trout begin to forage on salmon eggs, but the strength of the diet shift, and resulting growth rates, depends heavily on the abundance of salmon and the *in situ* productivity of the stream [21,22]. Interestingly, at the scale of entire watersheds, the number of salmon returning to spawn is relatively consistent among years due to fisheries management [23]. However, the number of salmon returning to individual streams can be extremely variable as a result of natural population dynamics that produce little synchrony in salmon abundance among streams and years within the same watershed [24]. Thus, at the scale of a single-stream, variability in salmon run size can make food resources highly variable among years. On the other hand, at larger spatial-scales, meta-population dynamics among the various sub-populations greatly increases the reliability of salmon subsidies to consumers at the basin scale [25,26], but it requires that individual fish move among tributaries to capitalize on the heterogeneity. Therefore, we characterized the movement patterns of resident fishes across a network of tributaries before and during salmon spawning to assess whether grayling and rainbow trout moved at spatial scales coarse enough to exploit the shifting foraging opportunities associated with salmon subsidies.

**Fig 1:**
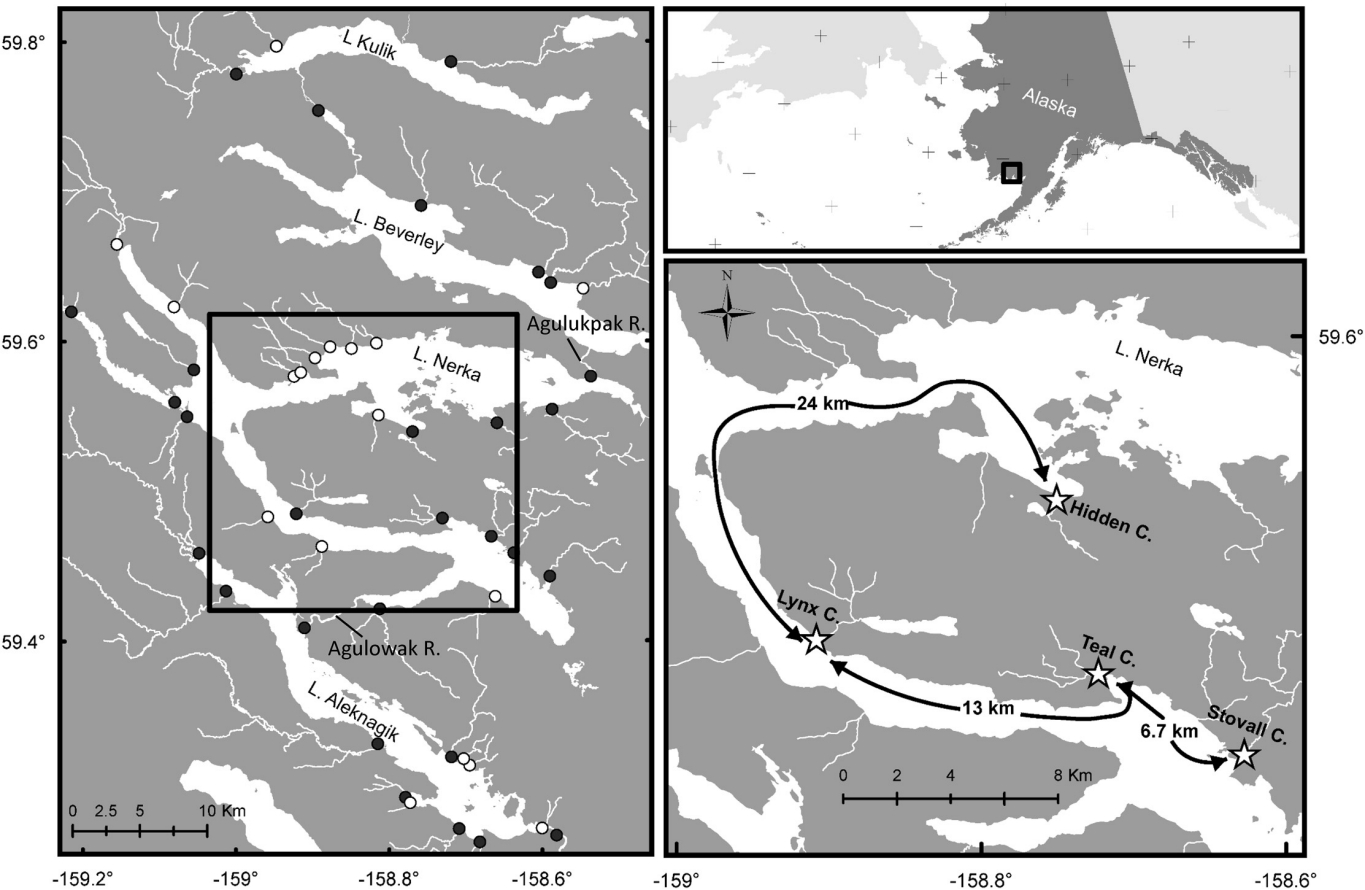
Map of the Wood River system, southwestern Alaska. Left panel denotes streams throughout the watershed where Arctic grayling and rainbow trout are either present (filled circle) or absent (open circle) during summer months. Lower-right panel denotes streams equipped with stationary PIT-tag antenna arrays. Note: this figure does not contain any copyrighted information.

We used individually tagged fish to investigate the intra-seasonal movement patterns of Arctic grayling and rainbow trout within a river basin in southwestern Alaska (Fig 1). Specifically, we monitored the movements of individual fish both within and among four tributaries, ranging in distance from 6.7–41 km apart, across two years. The objective of the study was to assess the functional connectivity of the four tributaries by measuring the seasonal timing and movement rates among individuals and species. This study provides one of the first-steps towards understanding not only the importance of connectivity to buffer bu individuals from heterogeneously distributed resources, but also their ability to potentially exploit this variation to increase demographic characteristics, such as foraging rates, growth, and survival [e.g., 13,14].

## Methods

### Study system

This study was conducted in the Wood River system (59° 34’37”N, 158° 48’25”W), which drains into Bristol Bay in southwestern Alaska. The Wood River basin has a total drainage area of 3590 km^2^ and consists of five large, deep, oligotrophic lakes that are fed by numerous tributaries and connected by small rivers. Our focal study system consisted of four tributaries (Hidden, Lynx, Teal, and Stovall creeks) that flow into Lake Nerka, which is the largest lake in the system, located midway up the river network (Fig 1). While grayling and rainbow trout inhabit dozens of tributaries across the Wood River watershed (Fig 1), our study streams were chosen based on (1) the suitability of habitat for resident fishes, (2) their geographic distribution across the lake, and (3) our ability to successfully monitor movements (specifically the width and depth of some streams were too large for our monitoring equipment). These four tributaries are 3^rd^- to 4^th^-order streams with average wetted widths of 4.5–6.5 m and have approximately 1–5 km of total stream length used by resident fishes.

Arctic grayling and rainbow trout were chosen as our focal species because they typically comprise >95% of the resident stream fish biomass in these streams. Though dominant, grayling and rainbow trout are part of a larger stream community consisting of Arctic char (*Salvelinus alpinus*), three-spined stickleback (*Gasterosteus aculeatus)*, juvenile coho *(O*. *kisutch)* and sockeye salmon, and coastrange (*Cottus cognatus)* and slimy (*C*. *aleuticus)* sculpin. Additionally, anadromous sockeye salmon spawn annually in streams and rivers that are inhabited by resident fishes. Although adult sockeye salmon are present throughout the river basin from mid-July through late-October, individual populations of salmon are typically active on individual spawning grounds for two to four weeks, and their spawning timing is tightly linked to stream temperature [6,25] In our four study streams, adult sockeye salmon commence spawning during the last week of July to the first week of August and by the first week in September most salmon have completed spawning and died.

### Data collection

We began sampling grayling and rainbow trout in Hidden and Lynx creeks in 2007, Teal Creek in 2010, and Stovall Creek in 2011 (S1 Table). Each year, the streams were sampled every 10 to 30 days from mid-June through early-September. During each sampling event, resident fish were collected in a spatially continuous manner throughout the stream using a stick seine[22]. Across the five years and four streams, we implanted a representative size range of fish (S1 Fig) with a unique passive integrated transponder (PIT)-tag (full duplex, 134.2 kHz, 11.5 mm length, 2.1 mm diameter; Allflex-USA, Dallas-Fort Worth Airport, Texas). Fish were anesthetized using AQUI-S (eugenol) prior to tagging. In total, we tagged 39% (n = 4590) and 67% (n = 1260) of all captured rainbow trout and Arctic grayling, respectively. Due to our sampling effort and the natural distribution of fish, >86% of all fish were tagged in either Hidden or Lynx creeks (S1 Table). Tagged individuals ranged in fork length from 69 to 558 mm, but >87% of all fish were between 100–300 mm (S1 Fig), and therefore likely sub-adults [27]. During these resident fish surveys, we also visually monitored the abundance of adult sockeye salmon by wading the entire main stem of the stream. We frequented these streams every 23 days during late-July to get a precise estimate of when salmon began spawning.

In 2011, we installed stationary PIT-tag antenna arrays [13,28] at Hidden, Lynx, and Teal creeks to continuously monitor the intra-seasonal movements of Arctic grayling and rainbow trout. In 2012, we added an additional detection site at Stovall Creek (Fig 1). Each site was equipped with a pair of PIT-tag antennas, placed approximately 4 m apart, which allowed for the assessment of movement direction. Each antenna was connected to a single PIT-tag reader, which recorded fish movements by storing individual tag IDs, along with the time and date, and the antenna location. Antennas were positioned perpendicular to the stream flow (i.e., pass-through antennas) and placed approximately 100–200 m upstream of the stream mouth, at a location were the antennas spanned the entire stream channel. Antennas were installed as soon after the spring freshet as possible. In 2011, antennas were installed June 12–16 and removed September 10–12. In 2012, antenna arrays were installed June 9–10 and removed September 6–11. Antenna sites were checked every 4–8 days to exchange batteries and download data. During each visit we tested the functionality of the site by passing a test PIT-tag through each antenna approximately five times. If the reader did not detect the test tag each time, the readers were adjusted until properly functioning. Read range was always greater than the height of the antennas, which were never fully inundated.

As part of our pilot study on fish movement in 2010, we visually estimated the relative intra-seasonal densities of Arctic grayling and rainbow trout using daytime snorkel surveys in Hidden and Lynx creeks. The purpose of these surveys was to characterize the change in densities over the course of the summer, determine the relative abundance of both species, and provide an independent estimate of population-level movements. Sample units were demarcated in each stream in early-June 2010 (Hidden: n = 44; Lynx: n = 67). The cumulative length of the sample units comprised ~35–50% of the total length of each stream. However, the sample units consisted of almost all of the pools in each stream, which is the preferred habitat for both rainbow trout and Arctic grayling, along with a sub-sample of runs and riffles. Thus, a majority of the realized habitat was surveyed during each sample event. During each survey, a single snorkeler moved upstream through each unit, enumerating the fish by species. This was a highly effective method as stream visibility always exceeded the width and depth of the habitat units sampled. Each stream was surveyed every 10–21 days from mid-June through mid-September. In 2011, we duplicated these surveys, but did not add Teal or Stovall creeks due to low visibility in those streams due to high concentrations of dissolved organic matter. In 2012, periodic rain events increased stream flows, inhibiting several surveys, and therefore these data were not included. For each year, relative densities were estimated by dividing the total number of each species enumerated throughout all sample units by the total area of stream surveyed (i.e., number of fish m^-2^).

All sampling and collection methods were conducted and approved in accordance to guidelines set by the Institutional Animal Care and Use Committee (IACUC) at the University of Washington (permit number 412–01). Additionally, the Alaska Department of Fish and Game (ADFG) reviewed and approved all of our methods and issued us field sampling permits (permit numbers: 2010—SF2010-151; 2011—SF2011-115; 2012—SF2012-110). Permission to work in the Wood-Tikchik State Park was granted by the Alaska Department of Natural Resources.

### Data analyses

Intra-seasonal movement patterns by Arctic grayling and rainbow trout were assessed using fish that were observed in a stream, but had been tagged in a previous year of the study. By using only “returner” fish we decreased our sample size, but were able to monitor the entire intra-seasonal movements of each individual fish, and avoiding confounding our analyses with any short-term changes in behavior associated with tagging. In 2011, 59 grayling and 77 rainbow trout “returned” to one or more of our monitored streams, while in 2012, 121 grayling and 141 rainbow trout were detected returning. Of the returning fish, 71–95% were tagged in the previous year, which comprised 16–29% of all fish tagged the year prior (S1 Table).

Based on our study design, we were able to characterize movements into (i.e., immigrations) and out of (i.e., emigrations) streams using a set of decision rules. Briefly, we first classified all movements using antenna hits that had serial detections, and used the sequence of antenna detections to define the direction of the movement (i.e., an immigration = detection on the downstream antenna, followed by a detection on the upstream antenna). Serial detections were defined as detections on two adjacent antennas within a span of 15 minutes. We chose this threshold because it was the approximate maximum amount of time it took an individual to move through both antennas based on visual examination of the data. The remaining “single” antenna hits were classified as either an immigration or emigration using information based on where the fish was initially tagged, where the fish was previously detected, and if the single hit was between two serial hits of the same direction (e.g., a single hit between two serial immigrations was assumed to be an emigration). Over the two years, brown bears (*Ursus arctos*), inclement weather, and electronic issues resulted in some equipment failure and data loss. However, these failures were relatively rare. Across all sites and years, at least one antenna was functioning >96% of the time and both were working >83% of the time. Based on two independent methods, our antenna detection efficiency averaged 83% and 90% (S1 File). Nonetheless, because our antenna detection efficiency was not perfect, our estimates of movement provide a conservative measure of the realized movement tendencies of these fishes.

To characterize movements we analyzed data both at individual sites as well as among streams. We began by calculating the elapsed time between consecutive detections, and thus, could determine the amount of time individuals spent either in the stream before emigrating or spent outside of a given stream before immigrating back. While we could classify the known location of a fish if it was detected immigrating into one of our four monitored sites, if a fish emigrated from a monitored site it resulted in one of three types of movements. First, if a fish left a particular site and was detected at another stream some time later, this emigration was defined as an inter-(among) stream movement. We calculated the total distance swam by each fish by enumerating the number of inter-stream movements within a given year and multiplying it by the respective distances between each of the sites. Hypothetically, if given sufficient time, a fish could have visited other streams where we did not have PIT-tag antennas between leaving its initial site and arriving at the next monitored stream. Therefore, the total number of sites visited, and total cumulative distance travelled, by each fish was a minimum estimate. Second, a fish could have emigrated from a stream and then immigrated back into the same stream some time later. We only defined that movement as an emigration if the fish left the stream for more than 24 hours. We based this threshold on the approximate minimum amount of time it took a fish to travel between two of our monitored sites (see results). Thus, if a fish did not leave a stream for >24 hours, it was assumed to have remained at that same site. Last, a fish could have left one of our monitored sites and was never detected again. Although we did not know where the individual went, we know that it emigrated to at least one additional site and therefore classified this as its final movement to an “unknown” stream and said it visited a “+” number of sites. While it was possible that a portion of these emigrating fish that we assigned a “+” movement died, opposed to immigrating to a new tributary, we assumed that mortality was negligible given that (1) there were few predators in Lake Nerka capable of eating the size of fish we studied, and (2) recreational fishing for rainbow trout and Arctic grayling was almost non-existent on Lake Nerka. Nevertheless, these individuals were still detected emigrating from one of our monitored tributaries. Therefore, these movements were at minimum a directed movement to another tributary whether the individual survival the trip or not. Movement data were summarized by species, stream, and year. If there were no significant difference among groupings or sample sizes were small (n < 30), data were lumped. Program R was used for all analyses [29].

## Results

**Fig 2:**
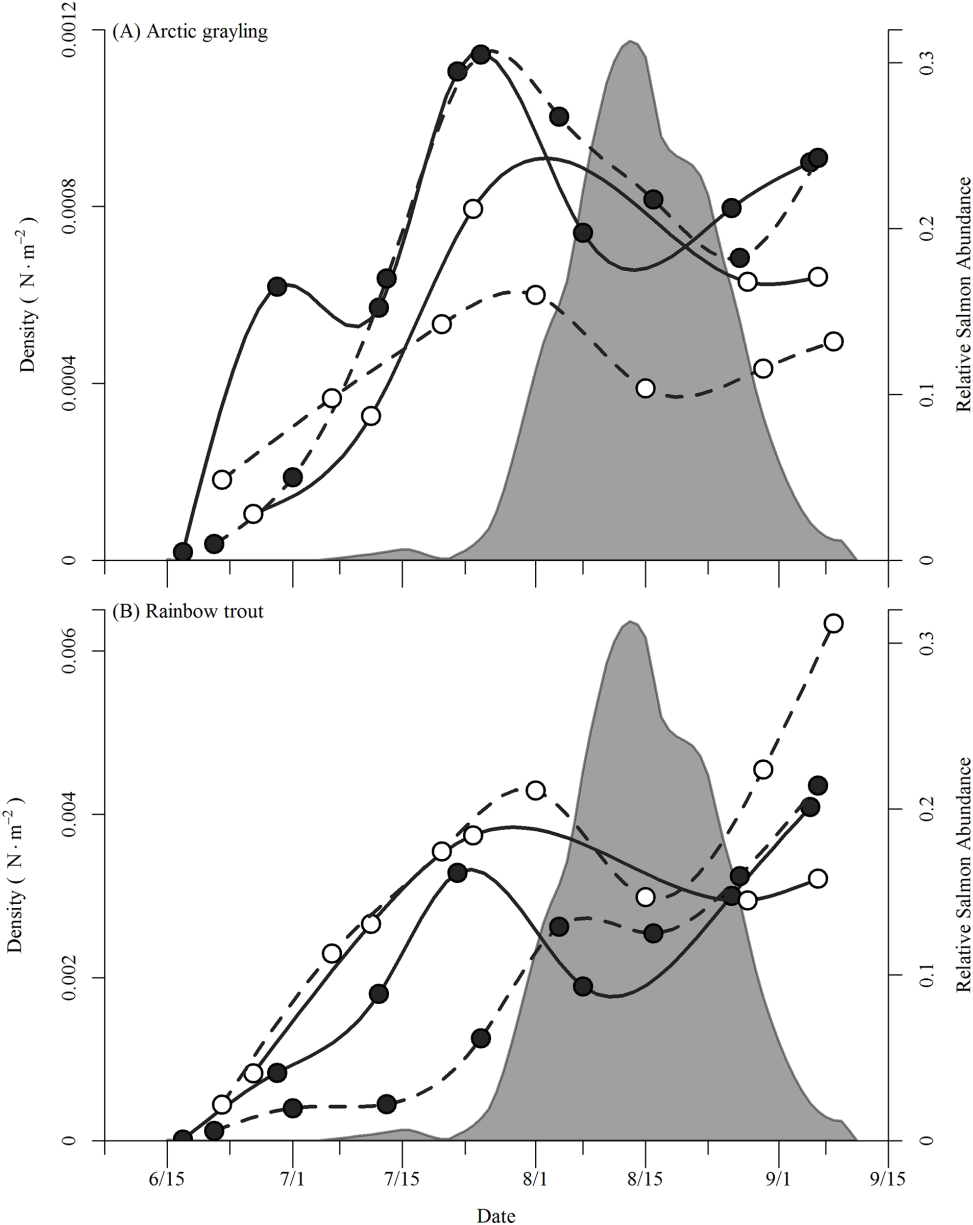
Density (N · m^-2^) of (A) Arctic grayling and (B) rainbow trout. in Hidden (filled circle) and Lynx (open circle) during 2010 (solid line) and 2011 (dashed line) estimated via snorkel surveys. The shaded area is the relative abundance of sockeye salmon averaged for both streams and years.

We observed strong seasonal shifts in the densities of resident fish observed during snorkel surveys at Hidden and Lynx creeks (Fig 2). Both streams held few fishes at the beginning of the growing season, during mid- to late-June, averaging (± SD) 14.2 ± 10.9 Arctic grayling and 84.1 ± 111.3 rainbow trout per stream kilometer. Over the following three to five weeks, densities quickly rose, and by late-July to early-August densities had increased 6 to 12-fold (159.6 ± 36.6 Arctic grayling and 545.2 ± 230.8 rainbow trout per stream kilometer). By late-August to early-September, densities of grayling remained at similar levels observed in late-July (130.7 ± 25.3 km^-1^) while rainbow trout densities reached their highest levels (772.9 ± 261.9 km^-1^).

**Fig 3:**
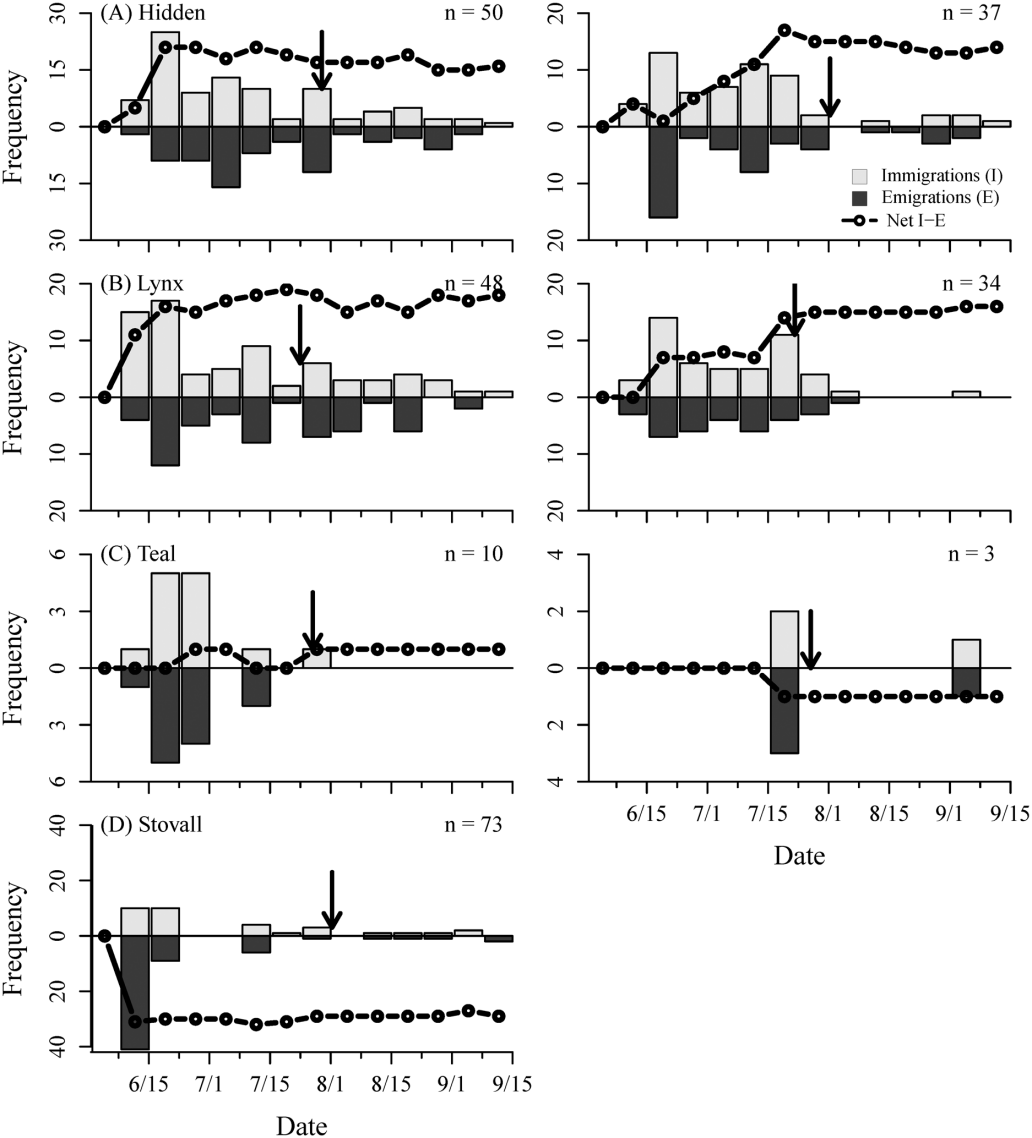
Immigrations (gray bars), emigrations (black bars), and the cumulative sum of immigrations and emigrations (black line) of PIT-tagged Arctic grayling. during 2012 (left) and 2011 (right) in (A) Hidden, (B) Lynx, (C) Teal, and (D) Stovall creeks. The number (n) on each panel denotes the total number of individual fish and the vertical arrow is the date of sockeye salmon entry.

**Fig 4:**
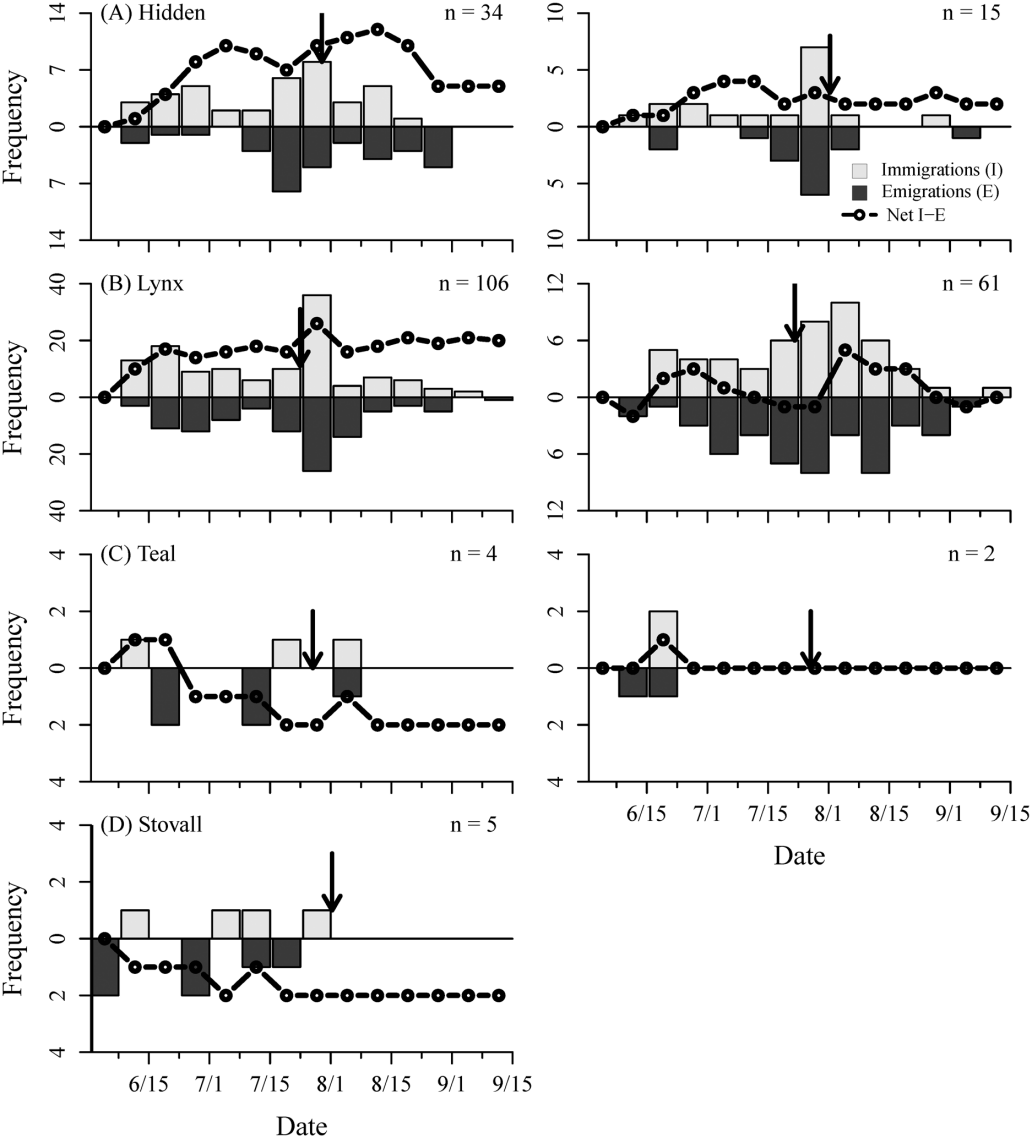
Immigrations (gray bars), emigrations (black bars), and the cumulative sum of immigrations and emigrations (black line) of PIT-tagged rainbow trout. during 2012 (left) and 2011 (right) in (A) Hidden, (B) Lynx, (C) Teal, and (D) Stovall creeks. The number (n) on each panel denotes the total number of individual fish and the vertical arrow is the date of sockeye salmon entry.

The observed changes in stream fish densities from snorkel surveys were corroborated by individual movements of PIT-tagged fish. Using our stationary antenna arrays, we detected an overall net influx of both Arctic grayling and rainbow trout in Hidden and Lynx creeks. Individuals began entering streams in mid-June and reached their highest abundances by late-July to early-August (Figs 3 and 4 –subpanels A and B). Though, unlike the snorkel surveys, which only could detect the relative change in population level abundance, our movement data revealed high rates of immigration and emigration (i.e., turnover) by individuals at each of our monitored sites. On average, 77 ± 14% of all individual grayling and 72 ± 15% rainbow trout emigrated (see methods for definition) from a stream at least once and, overall, for every 1.11 immigrations there was 1 emigration. Fish at Teal and Stovall creeks displayed similar behaviors to fish at Lynx and Hidden creeks (Figs 3 and 4 –subpanels C and D), but sample sizes were small, and therefore overall patterns were less discernable. The one exception was grayling at Stovall Creek, where we detected a large number of individuals early in the season (n = 73), but a net efflux.

**Fig 5:**
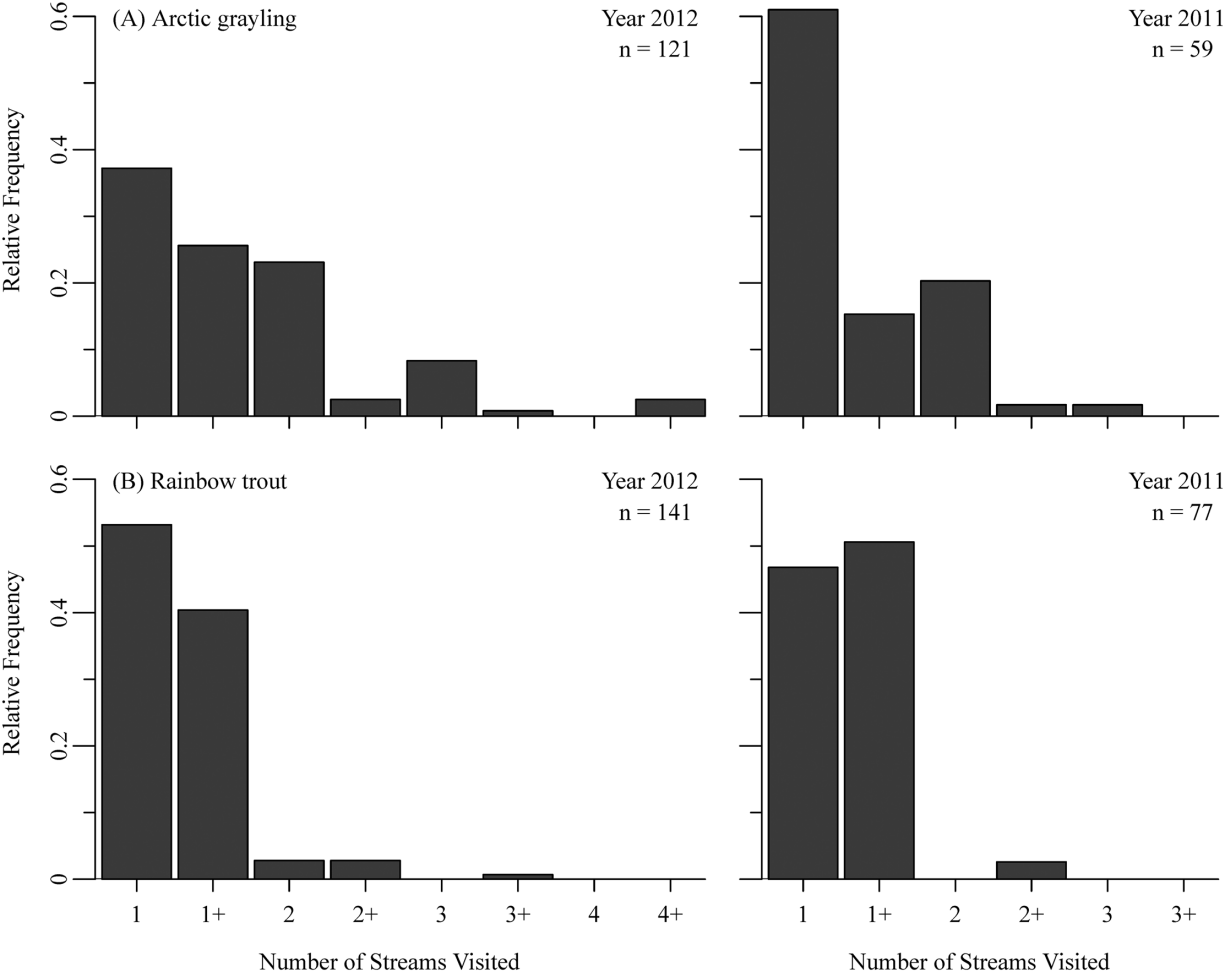
Relative number of streams visited by (A) Arctic grayling and (B) rainbow trout. in 2012 (left) and 2011 (right). Fish that visited a “+” number of streams (e.g., 1+) were last seen leaving a site and were not detected again in that year. Note: fish could have visited a site more than once (e.g., Hidden->Lynx->Hidden), but only unique visits were counted.

Individual movements were concentrated from mid-June through July (Figs 3 and 4). Overall, 90 ± 9% of all movements (i.e., immigrations and emigrations combined) by Arctic grayling occurred prior to the movement of spawning sockeye salmon into all four streams, despite this time period consisting of only 40–45% of the total monitoring period (proportion test; χ^2^ = 416.6, p < 0.001). Similarly, 81 ± 15% of all movements by rainbow trout across all sites occurred before August 1^st^ (χ^2^ = 161.7, p < 0.0001). While grayling and rainbow trout exhibited similar timing of their movements, they differed in their relative use of our monitored sites (Table 1; Fig 5). Combining both years, 33% of all grayling were detected at two or more of the monitored sites during a single summer, while only 5% of rainbow trout were detected making at least one inter-stream movement. However, of the individuals that were only detected at one of the monitored sites within a given summer, 32% of grayling and 46% of rainbow trout had to have immigrated to at least one additional non-monitored site, as they were last seen emigrating from a stream and were never detected again that year. Therefore, a minimum of 54% of grayling and 49% of rainbow trout used two or more streams across the river network during the summer foraging period in 2011 and 2012.

**Table 1:** Movement rates of individual Arctic grayling and rainbow trout among streams. Rates are defined as the number of movements between an individual stream divided by the total number of movement to other streams for each species. Fish moving to "unknown" streams are individuals last seen leaving a site and were not detected again in that year (either re-entering the same site or emigrating to another site). Fish moving "from" and "to" the same stream (e.g., Hidden to Hidden) are individuals that were last detected entering that stream; however, these fish could have immigrated from another site or left a stream and came back, but were not detected at another site. Movement rates were similar between 2011 and 2012, and thus combined.

			To Stream				
Species	From Stream	Sample size (N)	Hidden	Lynx	Teal	Stovall	Unknown
Arctic grayling	Hidden	98	0.48	0.21	0.00	0.05	0.26
	Lynx	94	0.31	0.47	0.04	0.00	0.18
	Teal	13	0.31	0.31	0.08	0.23	0.08
	Stovall	76	0.05	0.32	0.04	0.22	0.37
Rainbow trout	Hidden	49	0.31	0.06	0.00	0.00	0.63
	Lynx	171	0.04	0.39	0.01	0.00	0.56
	Teal	6	0.00	0.17	0.17	0.17	0.50
	Stovall	5	0.00	0.00	0.00	0.00	1.00

**Fig 6:**
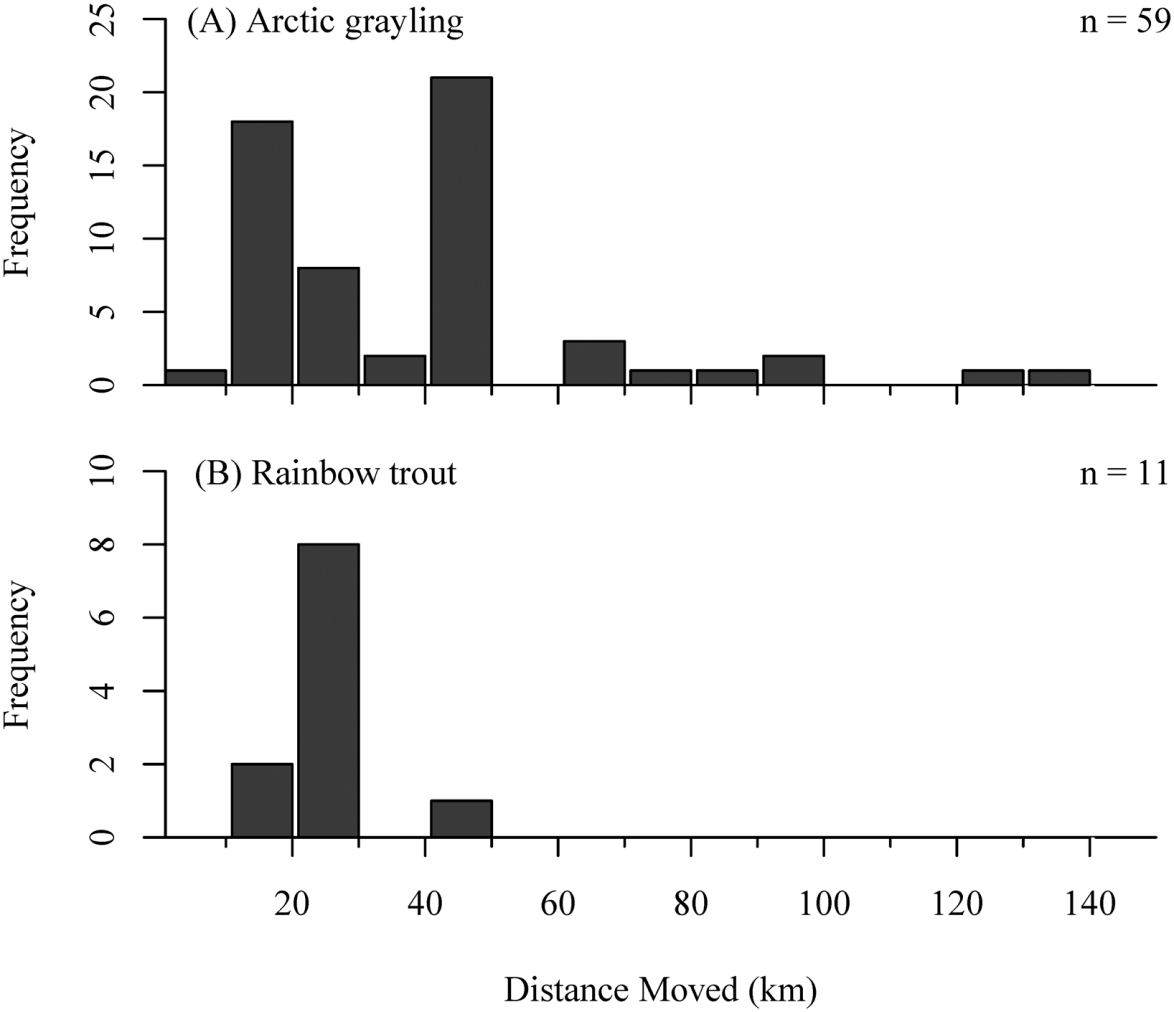
Minimum cumulative distance (km) of inter-stream movements by individual (A) Arctic grayling and (B) rainbow trout. for years 2011 and 2012 combined. Note: these distance estimates only include fish moving among monitored sites.

Over the course of a single summer, we observed a wide range of total distances travelled by individual fish. We observed Arctic grayling moving as little as 6.7 km between Teal and Stovall creeks, the minimum detectable distance, to as far as 130 km (from Stovall → Lynx → Hidden → Lynx → Hidden → Stovall over a 45 day period). Overall, the total minimum cumulative distance an individual Arctic grayling moved among streams in a single summer averaged 41.3 ± 31.8 km (Fig 6a). Among stream movements by individual rainbow trout ranged from 13 to 48 km, with an overall average of 24.8 ± 8.4 km (Fig 6b). The total distances swam between species were not significantly different in either 2011 (Wilcoxon sign-ranked test; p = 0.16) or 2012 (p = 0.57).

**Fig 7:**
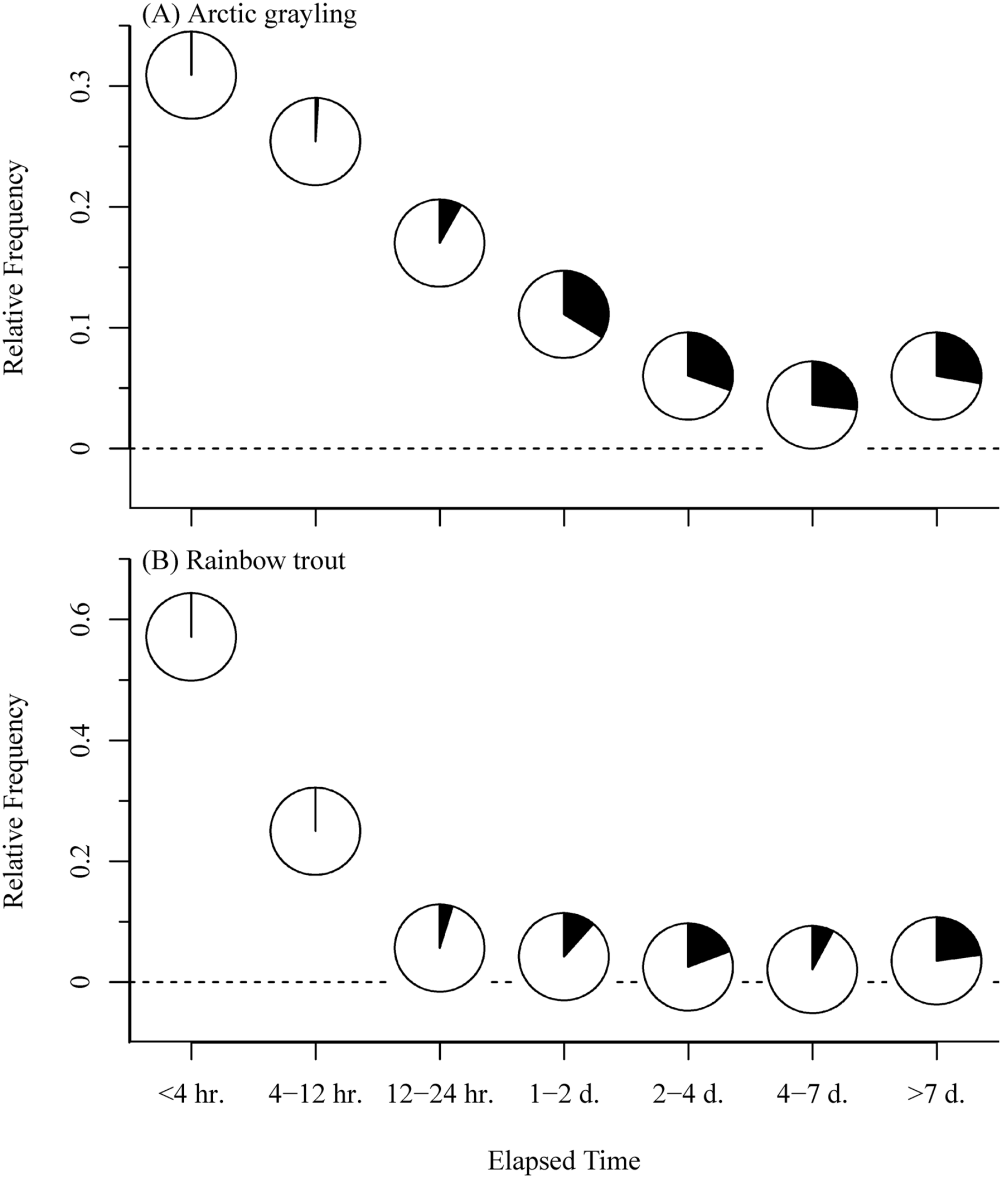
Relative frequency of transit time between movements. as a function of whether the movement was to a new stream. Transit time was calculated as the time elapsed between a downstream (emigration) and upstream (immigration) movement for (A) Arctic grayling and (B) rainbow trout. The black portion of the pie represents the relative proportion that were inter-stream movements (i.e., the white portion represents fish that emigrate from a stream and then immigrated back into the same stream, but were not detected at another site between those two movements).

**Fig 8:**
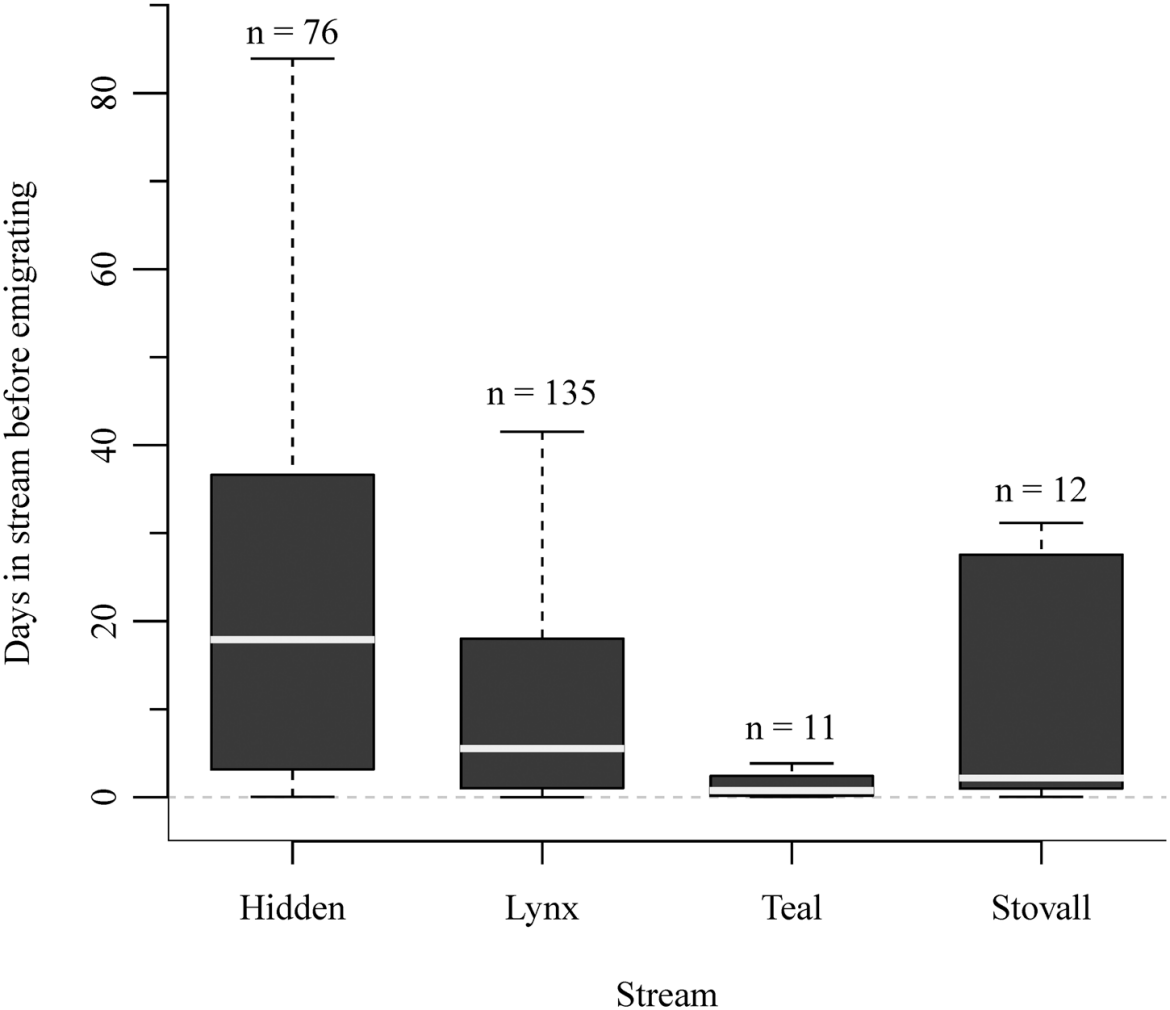
Duration of time (days) individual fishes spent in a stream prior leaving and immigrating to another stream. The light gray line in the box is the median; the lower and upper edges of the box are the lower and upper quartiles (25th and 75th percentiles, respectively); the lower and upper edges of the box are the lower and upper quartiles (25th and 75th percentiles, respectively; the smallest and largest observations (in the range not considered as outliers) are shown as the error bars. Data for Arctic grayling and rainbow trout collected in 2011 and 2012 were combined as there were no significant difference in duration of time between species or year.

Individual Arctic grayling and rainbow trout displayed a wide range of variation in the elapsed time between emigrations (Fig 7) and immigrations (Fig 8). For both species, the majority of emigrations (i.e., time between consecutive downstream and upstream movements) lasted <12 hours, and of these, <1% were movements among study sites (Fig 7). As the elapsed time of an emigration increased to >1 day, on average, 31% and 16% of all emigrations were detected among-stream movements for Arctic grayling and rainbow trout, respectively. Once an immigration had occurred, individuals spent anywhere between 12 minutes to 85 days in the stream before emigrating to another monitored site (Fig 8). Although there was no significant difference in the elapsed time of an immigration between species and years, we did detect a significant difference between some streams. Specifically, fish remained in Hidden Creek for a median (± SD) of 17.9 ± 20.6 days, which was statistically greater than the median time in Lynx (5.5 ± 18.6 days; Kruskal-Wallis multiple comparison, p = 0.003) and Teal creeks (0.74 ± 3.1 days; p = 0.0001), but not Stovall Creek (2.2 ± 31.5 days; p = 0.166) likely due to the small sample size and large individual variation at Stovall. All other pair-wise comparisons were statistically indistinguishable (p > 0.05). This wide range of variation in residency among sites and individuals was exemplified by a single Arctic grayling in 2012. On June 23, it immigrated to Hidden Creek and remained for ~10 days. It then emigrated, entered Lynx creek followed by Teal Creek, remaining in each stream for approximately 2 hours each, before moving to Stovall Creek, and staying for about 2 days. On July 12 it left Stovall, returning to Lynx Creek on July 14, and remaining for approximately eight hours before emigrating, after which it was never detected again. Over the course of this three week period, the fish had moved a minimum of 60.7 km.

## Discussion

Our data demonstrate that Arctic grayling and rainbow trout exhibit kilometer-scale, intra-seasonal movements between tributaries of a river basin during the summer growing season, which occur in addition to pronounced inter-seasonal changes in habitat use. In mid- to late-June, sub-adult resident fishes began immigrating into previously unoccupied streams and over the following month relative densities increased 6–12 fold (Fig 2). Had we only used snorkel surveys to characterize habitat use these increases in fish density would have appeared to only be a population-level shift in habitat use between overwintering and summer foraging habitats. However, by monitoring the movement of individuals, we found that while there was typically an overall net influx of grayling and rainbow trout into streams in June and July, there was simultaneously high rates of immigration and emigration, producing substantial turnover in the identity of individuals comprising the population (Figs 3 and 4). Additionally, by monitoring movements at several tributaries across Lake Nerka, we also detected movements among streams that occurred throughout the growing season (Figs 5 and 6, Table 1). Therefore, large-scale movements may not only be necessary for individuals to complete the different stages of their life-cycle, but may be an important component of fish behavior as individuals navigate the heterogeneity of riverscapes.

Over the course of three months, we observed both Arctic grayling and rainbow trout moving among streams separated by 6.7–41 km. By using paired antennas at the mouth of each stream, we were able to assess the directionality of movements and determine the location (i.e., in a monitored stream or not) of all fish through time. Therefore, although an individual may have left one of our monitored streams and never detected again, we can use other knowledge of the river network to have some idea of where the fish may have immigrated. For example, while we know from radio-tracked rainbow trout adults that there is little movement among lake basins within the Wood River basin [30], there are more than 20 other streams within the watershed of Lake Nerka, 11 of which are used by grayling and rainbow trout during the summer foraging period (Fig 1; Alaska Salmon Program, *unpublished data*). Specifically, some resident fishes are likely moving to the Agulowak and Agulukpak rivers, which connect Lake Nerka to Lake Aleknagik and Lake Beverley. These two rivers offer high quality habitat and foraging opportunities, but are difficult to sample due to their size. Additionally, we can use information on among-stream travel time to estimate whether an individual could have visited an unmonitored site between consecutive emigrations and immigrations (Fig 7). For example, we detected a high frequency of emigrations lasting <12 hours, but few produced among stream movements that we detected. While movement distance will obviously influence the time needed for an individual to travel between two streams, most inter-stream movements took >12 hours. Thus, we can postulate that if an individual left one of the monitored sites for >24 hours before returning, it had the opportunity to have moved to an unmonitored site and back during that time. One assumption we are making here is that fish are only using Lake Nerka as a movement corridor and not actually for foraging or refuge. While we cannot reject this possibility, it is unlikely given that grayling and rainbow trout are rarely caught in lake habitat of the Wood River system by either anglers or during extensive beach seine sampling for juvenile salmon (Alaska Salmon Program *unpublished data*). Regardless, fish were using disparate habitats separated by up to tens of kilometers over a course of days to weeks during the summer growth period.

Fish may exhibit high rates of movement to capitalize on spatially and temporally heterogeneous early-season growing opportunities. The majority of movements by Arctic grayling and rainbow trout occurred in early summer, with few detected after August 1^st^. Fish in temperate streams can be in relatively poor body condition following the long and energetically challenging winter period [31,32]. Resident fishes feed on benthic and terrestrial invertebrates during June and July prior to the seasonal influx of high-quality salmon-derived resources [19]. Similar to salmon abundance, *in situ* productivity varies among streams. For example, the densities of benthic invertebrates are approximately 5–7 fold higher in Hidden Creek relative to Lynx Creek [33] and may explain why fish remained in Hidden Creek significantly longer before emigrating (Fig 8). But, resident fishes, and their associated movements, are most certainly influenced by a range of biological and environmental conditions [34–36]. Specifically here, it is likely that the movements by grayling and rainbow trout were at least partially influenced by foraging opportunities afforded by spawning sockeye salmon, as has been suggested by other studies [37–39].

The relative decrease in resident fish movement activity coincided with the occupation of streams by spawning sockeye salmon. Streams with spawning salmon offer high quality foraging opportunities for stream fishes [20] and previous research has shown that ration sizes and energy intake of grayling and rainbow trout can increase 400–600% after salmon arrive [19,21]. However, the ration sizes and growth rates of these fishes are heavily influenced by the relative abundance of salmon, which can vary up to 10–20 fold among years for individual streams [22,24]. Given that rainbow trout and grayling have the capacity to move among streams, we initially anticipated that there would be more resident fish movement during the peak of salmon spawning activity, especially if there were relatively few spawning salmon, such as in Hidden and Lynx in 2011 and 2012 [22]. We offer two explanations for why resident fish stop moving among streams during the salmon spawning season. First, the opportunity cost associated with leaving a stream that already has salmon, regardless of the density, may be too high to offset the potential gains of moving to a stream with an unknown amount of salmon. Second, some grayling and rainbow trout may be prospecting a range of potential foraging sites across the watershed during early summer and potentially using that information to inform current and future movement decisions [40–42]. While the mechanisms causing grayling and rainbow trout to move large distances are unclear, a large fraction of each population were documented moving among heterogeneously distributed habitat patches.

Our study is one of only a handful to document long-distance, intra-seasonal movements by stream-dwelling fish (e.g., [39,43]). In terrestrial systems, there is accumulating evidence that large-scale movements are essential for animals to achieve positive energy balance in patchy environments [15–17]. Specifically, it is increasingly recognized that although growth potential follows strong seasonal trends, there may be substantial variation at shorter time-scales [44–46], and thus the need for individuals to integrate across this variability. However, our understanding of how resource variation influences fishes and their related movements at shorter time-scales (days to weeks) but at larger spatial scales remains unclear. A key question is whether the lack of studies on large-scale resource tracking in fish reflects a lack of occurrence in nature, or a lack of detection by scientists.

It is interesting to consider why movements at larger spatial scales are rarely documented in freshwater fishes. One explanation is that the range of observed movement distances will directly depend on the interplay between the finest spatial and temporal resolution studied (grain) and the size of the entire study area or duration ([12],extent; [47]). As Gowan et al. [48] highlighted, movement studies can be biased against detecting long-distance movements because of a failure to monitor over large areas. Second, movement studies often seek to further study historically recognized patterns of movement (e.g., smolt and spawning migrations) rather than detect new ones. Also, available methods (where you have to place detection sites where you anticipate movement) often inhibit our ability to stumble upon new movement behavior. Specifically, fish ecologists lack the continuous high scope (i.e., high resolution and large extent) movement data provided by GPS collars, which continually reveal new movement behavior in birds and mammals (e.g., [49]). Last, movement studies are routinely conducted in fragmented river systems possibly biasing our understanding of movement patterns and connectivity in intact river networks. While impassable barriers are known to directly inhibit movement [50–52], it is possible that they also indirectly affect the range of observed movement distances. Similar to studies that have shown that recreational fisheries indirectly decrease the overall growth rate of populations by harvesting the most aggressive, fast-growing individuals [53], barriers could potentially remove the most mobile individuals while the more sedentary are more likely to persist [54]. Further, alteration of habitat, such as semi-permeable barriers on terrestrial landscapes, can inhibit the ability of mobile consumers to effectively track resource heterogeneity across landscapes [55]. Therefore, movement studies conducted in impacted watersheds may not fully represent the historic movement capacity of fish populations. One promising area of future research will be to model how individual fish movements interact with varying degrees of spatially and temporally heterogeneous resources, and various scenarios of habitat connectivity, to influence the persistence, abundance, and productivity of resident fish populations.

In summary, this study showed that a high proportion of individual resident fishes used multiple streams across a river network during the summer foraging period. Although we did not directly measure the factors controlling the movement of individuals, the spatial and temporal scale at which the movements occurred provide insight to the spatial scales that heterogeneously spaced resources matter to these fishes. Previous studies have highlighted the importance of maintaining connectivity among complementary habitats for individuals to complete the different stages of their life-cycle [7,9,12]. Our results highlight that individuals may also need to move large distances among streams and habitats to capitalize on patchily distributed foraging opportunities. Therefore habitat fragmentation and homogenization may have even farther reaching effects on the productivity and long-term persistence of stream-dwelling fish populations than previously recognized.

## Supporting Information

S1 Table Distribution of PIT-tags deployed and returned among species, sites, and years. Number of Arctic grayling and rainbow trout tagged and returned (in subsequent years) by year and stream. Only "returning" fish were used in movement analyses.
(DOCX)

S1 Fig Length-frequency histograms of all hand-captured (light gray) and tagged (black) Arctic grayling (left) and rainbow trout (right). in (A) Hidden Creek, (B) Lynx Creek, (C) Teal Creek, and (D) Stovall Creek.
(TIF)

S1 File Antenna detection efficiency. (DOCX)
