# Supplementary material for: Inter-Tributary Movements by Resident Salmonids across a Boreal Riverscape
Source: PLoS One. 2015 Sep 17;10(9):e0136985. doi: 10.1371/journal.pone.0136985 (PMC4574770; doi:10.1371/journal.pone.0136985)
Supplement: S1 Table — Number of Arctic grayling and rainbow trout tagged and returned (in subsequent years) by year and stream. Only "returning" fish were used in movement analyses. (DOCX) [file pone.0136985.s001.docx]

|  | | | | | | | |
| --- | --- | --- | --- | --- | --- | --- | --- |
|  |  | Tagged | | |  | Returned | |
| Species | Stream | 2007-2009 | 2010 | 2011 |  | 2011 | 2012 |
| Grayling | Hidden | 132 | 128 | 122 |  | 38 | 51 |
|  | Lynx | 156 | 108 | 129 |  | 34 | 49 |
|  | Teal | 0 | 0 | 32 |  | 3 | 10 |
|  | Stovall | 0 | 0 | 37 |  | - | 73 |
|  | Total | 288 | 236 | 320 |  | 59* | 121* |
|  |  |  |  |  |  |  |  |
| Rainbow trout | Hidden | 344 | 111 | 200 |  | 15 | 34 |
|  | Lynx | 509 | 232 | 371 |  | 62 | 108 |
|  | Teal | 0 | 0 | 23 |  | 2 | 4 |
|  | Stovall | 0 | 0 | 0 |  | - | 5 |
|  | Total | 853 | 343 | 594 |  | 77* | 141* |
| * Note: Total does not equal the sum of all streams because some fish "returned" to multiple streams | | | | | | | |
