# Supplementary figures and images for: Inter-Tributary Movements by Resident Salmonids across a Boreal Riverscape
Source: PLoS One. 2015 Sep 17;10(9):e0136985. doi: 10.1371/journal.pone.0136985 (PMC4574770; doi:10.1371/journal.pone.0136985)

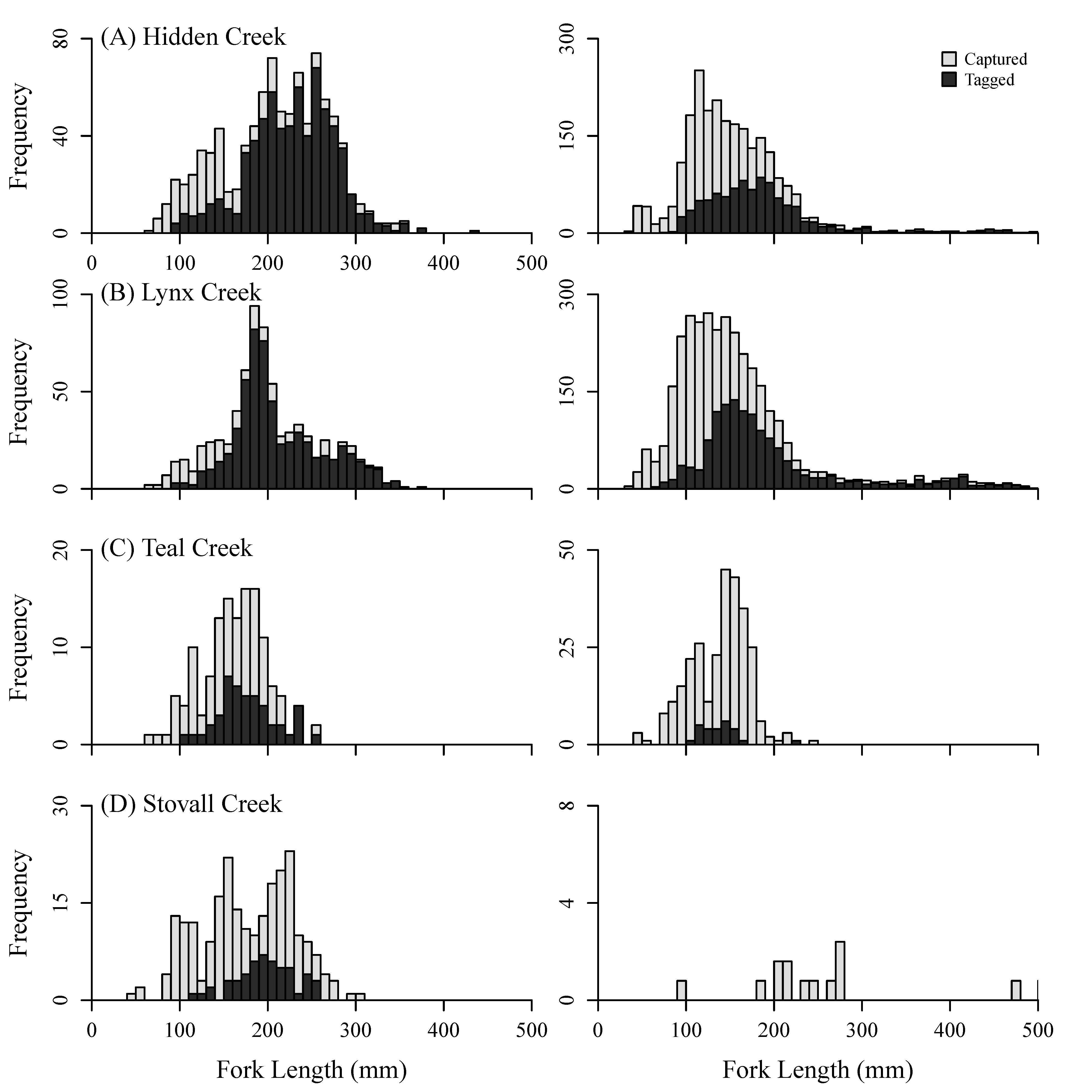

Supplement: S1 Fig — in (A) Hidden Creek, (B) Lynx Creek, (C) Teal Creek, and (D) Stovall Creek. (TIF) [file pone.0136985.s002.tif]
