# Supplementary material for: Inter-Tributary Movements by Resident Salmonids across a Boreal Riverscape
Source: PLoS One. 2015 Sep 17;10(9):e0136985. doi: 10.1371/journal.pone.0136985 (PMC4574770; doi:10.1371/journal.pone.0136985)
Supplement: S1 File — (DOCX) [file pone.0136985.s003.docx]

Antenna detection efficiency was assessed two ways. First, we calculated the percent of total antenna detections that were classified as serial detections. Second, we compared the known location of recaptured individuals from our stream seining data to the predicted location of each individual based on the antenna data. Over the two years of this study, a total of 8,442 antenna hits were classified as movements and overall 83.4% of the movements were serial detections (Table A; ranging from 63.8% in Lynx during 2012 to 98.5% in Stovall during 2012). During 2011 and 2012, we recaptured 363 Arctic grayling and rainbow trout “returners” in one of our four monitored streams. Overall, 90.1% of these recaptures were correctly classified as being in the stream they were captured in based on their antenna detection (Table B; ranging from 77.8% in Stovall during 2012 to 100% in Teal during 2011).

| Table A. Percent of antenna hits that were serial detection by stream, species, and year. | | | | | |
| --- | --- | --- | --- | --- | --- |
|  |  | Stream | | | |
| Species | Year | Lynx | Hidden | Teal | Stovall |
| Rainbows | 2011 | 87.2 | 86.5 | 76.5 | - |
|  | 2012 | 79.2 | 93.7 | 95.8 | 98.5 |
| Grayling | 2011 | 82.6 | 74.3 | 89.2 | - |
|  | 2012 | 63.8 | 89.4 | 96.4 | 90.7 |

| Table B. Percent of handheld capture locations that matched predicted locations from antenna data by stream and year. Sample sizes are denoted in parentheses. | | | | | | | | |
| --- | --- | --- | --- | --- | --- | --- | --- | --- |
|  | Stream | | | | | | | |
| Year | Hidden | | Lynx | | Teal | | Stovall | |
| 2011 | 84.2 | (133) | 94.5 | (69) | 100.0 | (7) | - | - |
| 2012 | 98.0 | (101) | 78.6 | (28) | 91.7 | (12) | 77.8 | (9) |
